# Screening of Metagenomic and Genomic Libraries Reveals Three Classes of Bacterial Enzymes That Overcome the Toxicity of Acrylate

**DOI:** 10.1371/journal.pone.0097660

**Published:** 2014-05-21

**Authors:** Andrew R. J. Curson, Oliver J. Burns, Sonja Voget, Rolf Daniel, Jonathan D. Todd, Kathryn McInnis, Margaret Wexler, Andrew W. B. Johnston

**Affiliations:** 1 School of Biological Sciences, University of East Anglia, Norwich Research Park, Norwich, United Kingdom; 2 Department of Genomic and Applied Microbiology & Göttingen Genomics Laboratory, Institute of Microbiology and Genetics, Georg-August-University Göttingen, Göttingen, Germany; University of Delhi, India

## Abstract

Acrylate is produced in significant quantities through the microbial cleavage of the highly abundant marine osmoprotectant dimethylsulfoniopropionate, an important process in the marine sulfur cycle. Acrylate can inhibit bacterial growth, likely through its conversion to the highly toxic molecule acrylyl-CoA. Previous work identified an acrylyl-CoA reductase, encoded by the gene *acuI*, as being important for conferring on bacteria the ability to grow in the presence of acrylate. However, some bacteria lack *acuI*, and, conversely, many bacteria that may not encounter acrylate in their regular environments do contain this gene. We therefore sought to identify new genes that might confer tolerance to acrylate. To do this, we used functional screening of metagenomic and genomic libraries to identify novel genes that corrected an *E. coli* mutant that was defective in *acuI,* and was therefore hyper-sensitive to acrylate. The metagenomic libraries yielded two types of genes that overcame this toxicity. The majority encoded enzymes resembling AcuI, but with significant sequence divergence among each other and previously ratified AcuI enzymes. One other metagenomic gene, *arkA*, had very close relatives in *Bacillus* and related bacteria, and is predicted to encode an enoyl-acyl carrier protein reductase, in the same family as FabK, which catalyses the final step in fatty-acid biosynthesis in some pathogenic Firmicute bacteria. A genomic library of *Novosphingobium,* a metabolically versatile alphaproteobacterium that lacks both *acuI* and *arkA,* yielded *vutD* and *vutE,* two genes that, together, conferred acrylate resistance. These encode sequential steps in the oxidative catabolism of valine in a pathway in which, significantly, methacrylyl-CoA is a toxic intermediate. These findings expand the range of bacteria for which the *acuI* gene encodes a functional acrylyl-CoA reductase, and also identify novel enzymes that can similarly function in conferring acrylate resistance, likely, again, through the removal of the toxic product acrylyl-CoA.

## Introduction

Acrylate is a well-known compound, largely due to its use as a major chemical feedstock for acrylyl polymers in paints and other products of the petrochemical industries. In natural environments, though, it only occurs in significant amounts in few, specific niches. This is because it is the product of a particular catabolic reaction, namely the cleavage of dimethylsulfoniopropionate (DMSP), a highly abundant (∼10^9^ tons produced per annum) osmoprotectant and anti-stress compatible solute that is made by many marine photosynthetic phytoplankton, macroalgal seaweeds and a few angiosperms [1]–[4]. When stressed, some of these algae can cleave their DMSP into the volatile dimethyl sulfide (DMS) plus acrylate, which may provide protection against further predation by zooplankton [5]. Also, the DMSP that is released when plankton die or are grazed can be catabolised by marine bacteria and some fungi, which produce enzymes that are generically termed ‘DMSP lyases’ which also yield acrylate plus DMS [1]. Of these two DMSP cleavage products, DMS has received more attention, because of its important environmental effects in the global sulfur cycle and, perhaps, on climate through its effects on cloud formation [6].

Nevertheless, acrylate itself is also important, at least locally. It is abundant in (e.g.) corals, which are massive sources of DMSP, made by the dinoflagellete *Symbiodinium* [7], [8] and/or their hosts [9]. Although at high concentrations and low pH, acrylate may harm some marine microbes [10], some bacteria thrive in the high concentrations around cells of the phytoplankton *Phaeocytsis* [11], and some can use it as a sole carbon source for growth [1], [12], [13]. Not surprisingly, therefore, the study of acrylate catabolism has been intimately tied up with the ability of bacteria to use DMSP as a substrate.

Recent genetic studies (reviewed in [3], [4]) have revealed striking diversity in the ability to catabolise DMSP. At least seven different “Ddd” enzymes that cleave DMSP, and which generate DMS as an initial product, have been described in different marine bacteria. Five of these (DddL, DddP, DddQ, DddW and DddY) yield acrylate as the C3 catabolite, but despite these similarities in their substrate and in the resultant products, these enzymes are in at least four very different polypeptide families. In many different bacteria, these “primary” *ddd* genes are clustered with other *ddd* genes that are variously involved in DMSP import, in downstream catabolic steps and/or in gene regulation.

DMSP is also subject to a different catabolic fate, in which it is demethylated to methylmercaptopropionate (MMPA) via a DmdA demethylase. This pathway occurs in two abundant marine alphaproteobacterial groups, the SAR11 clade and the Roseobacters [14], and yields neither DMS nor acrylate. However, methylthioacrylyl-CoA is a downstream product [15], [16], and it had been suggested that demethiolation of MMPA might also generate acrylate [17].

During the course of analysing the *ddd* gene clusters of several bacteria, we noted that most of these included a gene that we termed *acuI* (acrylate utilisation). This was first identified in *Rhodobacter sphaeroides* 2.4.1 [18], in which *acuI* is the central gene of an operon whose promoter-distal gene, *dddL,* encodes a lyase that cleaves DMSP into DMS plus acrylate [19]. The promoter-proximal gene, *acuR,* encodes a transcriptional regulator that represses the entire operon, unless relieved by the presence of acrylate, which is the co-inducer molecule. AcuI^−^ mutants were defective in converting ^14^C-labelled acrylate to ^14^CO_2_, and, strikingly, were hyper-sensitive to the inhibitory effects on growth of exogenous acrylate [18]. They were also defective for growth on 3HP as a carbon source [20], [21].

Genes that closely resembled *acuI* were found not only in the *ddd* clusters of different bacteria (including those for *dddD* in *Halomonas, dddY* in *Alcaligenes,* and *dddP* in *Candidatus* Puniceispirillum marinum) but we noted an *acuI-*like gene immediately 3′ of *dmdA* in nearly all Roseobacter strains, and that the expression of this *dmdA-acuI* operon is much-enhanced by growth of cells with acrylate [22].

The AcuI enzyme is in the MDR012 subgroup of the very large medium chain dehydrogenase/reductase (MDR) superfamily [23]. It was recently shown to be an acrylyl-CoA reductase, converting acrylyl-CoA to propionyl-CoA [20] with high specificity for acrylyl-CoA, and a very low K_m_ (<3 µM) for this reaction [21]. This could explain the phenotypes of AcuI^−^ mutants, including their increased sensitivity to acrylate, since these would accumulate acrylyl-CoA, a very active cytotoxic electrophile that attacks sulfhydryl groups [24]. One enzyme in particular, pyruvate formate lyase, is hyper-sensitive to acrylyl-CoA [25], perhaps explaining why acrylate was more toxic to *E. coli* growing in anaerobic than in aerobic conditions [26].

Taken together, these observations suggest that the *acuI* gene in these various *ddd* (and *dmdA*) clusters may have an adaptive, protective role, via the transformation of the very reactive acrylyl-CoA into the less harmful propionyl-CoA [22]. However, other observations have recently shown that AcuI-type enzymes must have more complex and wide-ranging functions than just this.

We noted [22] that the taxonomic distribution of bacteria with *acuI* homologues was widespread, but sporadic. Thus, there are several bacterial phyla (e.g. the Chlamydia and the Spirochetes to name but two) in which none of the genome-sequenced strains contain an *acuI-*like gene and others in which only some contain it. For example, only one strain (*Gallibacterium anatis* UMN179) in the Pasteurellales Order of gammaproteobacteria has an AcuI-like polypeptide; other species, including the important pathogens *Haemophilus*, *Pasteurella* and *Mannheimia*, do not. This diverse, sporadic distribution implies that *acuI* has been subject to several rounds of horizontal gene transfer.

Furthermore, AcuI homologues occur in many bacteria (including *E. coli*) that do not, as far as we know, normally encounter acrylate or DMSP in their natural environments [22]. Thus, mutations in the *E. coli acuI* gene (previously termed *yhdH*) cause marked increased in acrylate sensitivity, a phenotype that could be corrected by cloned *acuI* from a range of bacteria, not only those with known connections with DMSP or acrylate [22]. Further, the *in vitro* properties of the *E. coli acuI* gene product closely resembled those of the *Rhodobacter* version (see above) in terms of its affinity and specificity for its acrylyl-CoA substrate [21]. Very recently, the enzymatic mechanism of two MDR012 members (including *E. coli* YhdH) was determined, with a wholly novel and unexpected intermediate being identified, comprising a covalent ene adduct between the NADPH coenzyme and the substrate [27].

In addition to AcuI, three other types of acrylyl-CoA reductase have been described. The first of these is an NADPH-dependent acrylyl-CoA reductase, forming part of the autotrophic CO_2_ fixation cycle [28] in some crenarchaea. Like AcuI, this enzyme is in the MDR superfamily, but it has no significant sequence similarity with characterised AcuI proteins. Secondly, the C-terminal domain of a propionyl-CoA synthase from the anoxygenic photosynthetic bacterium *Chloroflexus aurantiacus* is an NADPH-dependent enoyl-CoA reductase that reduces acrylyl-CoA to propionyl-CoA, also in the 3HP cycle of autotrophic CO_2_ fixation [29]. Finally, a different, NADH-dependent acrylyl-CoA reductase in *Clostridium propionicum* was reported to reduce acrylyl-CoA to propionyl-CoA [30].

The genomes of many other bacteria lack genes for any of these acrylyl-CoA reductases, so we set out to determine if we could isolate other genes, which when cloned, might confer acrylate resistance. To do this, we took a functional metagenomic approach [31]–[33], which sampled a wide portfolio of genomes, and which did not depend on homologies with known polypeptides, or on culture-dependent enrichments. By such means, we obtained various cosmids and plasmids that contained cloned DNA from different metagenomes and bacterial genomes which corrected the extreme sensitivity of the AcuI^−^ (YhdH^−^) *E. coli* mutant.

## Results

### Screening Metagenomic Libraries for Genes that Restore Acrylate Resistance to an *E. coli* AcuI^−^ mutant

**Figure 1:**
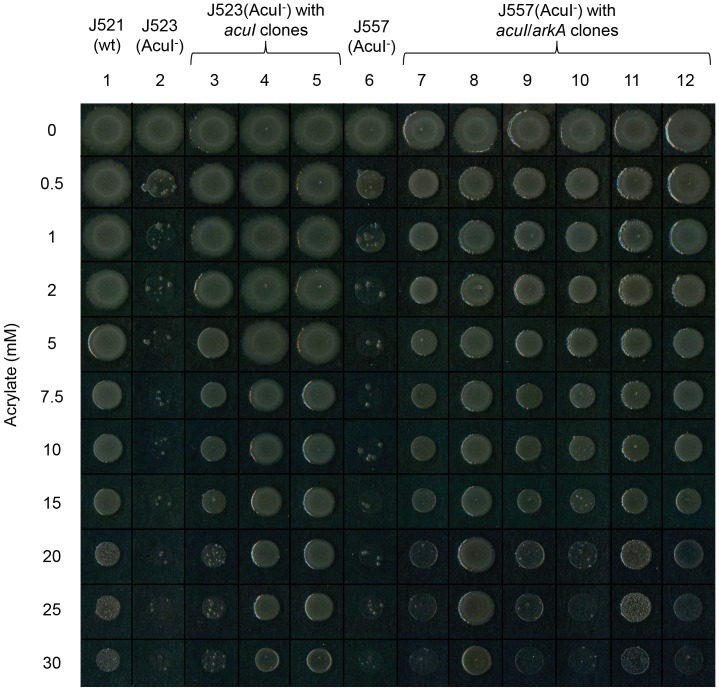
Correction of acrylate sensitivity of *E. coli* AcuI^−^ mutant by metagenomic library clones. Aliquots (10 µl) of *E. coli* cells were spotted on LB agar, containing increasing amounts of acrylate, shown as mM concentrations at left of panel. Plates were incubated at 37°C overnight before recording growth, shown in this composite. The strains used were as follows: Lane 1, wild type *E. coli* J521; Lanes 2 and 6, AcuI^−^ mutants J523 and J557 respectively; Lane 3, J523 corrected by *E. coli acuI* cloned in pET21a (pBIO2011); Lanes 4 and 5, J523 corrected by metagenomic *acuI-*like genes cloned in pLAFR3-based cosmids pBIO2079 and pBIO2081 respectively; Lanes 7, 8, 9, 10, 11, J557 corrected by metagenomic *acuI-*like gene cloned in pCR-XL-TOPO–based plasmids pBIO2151, pBIO2152, pBIO2153, pBIO2154 and pBIO2155 respectively; Lane 12, J557 corrected by metagenomic *arkA-*like gene cloned in pCR-XL-TOPO-based plasmid pBIO2160.

To identify microbial genes that conferred acrylate resistance, we exploited the marked acrylate sensitivity of the *E. coli* AcuI^−^ (YhdH^−^) mutant, which was used as the recipient strain with donor cultures of *E. coli* that harboured plasmids or cosmids that comprised individual metagenomic libraries. Selection was made for acrylate-resistant (Acr^R^) transconjugants on LB medium containing 2 mM acrylate, a concentration on which wild type *E. coli* grows well but which completely inhibits the *acuI* mutant [22] (Figure 1).

Two, pre-existing groups of metagenomic libraries were used (see Table S1 for details). In one set, large-insert (>25 kb) DNA fragments, isolated directly from bacteria in a waste-water treatment plant, had been cloned into pLAFR3, a wide host-range cosmid vector [34]; these cosmids were transferred, *en masse,* by triparental conjugational mating into the Rif^R^ *E. coli acuI* mutant J523. The other libraries comprised smaller inserts, obtained from several different environments (Table S1), cloned in the high copy number plasmid pCR-XL-TOPO; these were electroporated into the Spc^R^ *E. coli acuI* mutant J557. The entire inserts of the pCR-XL-TOPO-based plasmids were then sequenced; with the cosmid-based clones, the relevant region was first localised by identifying smaller fragments which, when sub-cloned into pBluescript, conferred an Acr^R^ phenotype.

This procedure yielded a total of 8 different metagenomic cosmids or plasmids that conferred an Acr^R^ phenotype. The introduction of each of these conferred tolerance to acrylate concentrations that were at least 10-fold higher than the 1 mM that completely blocked growth of the *E. coli acuI* mutant (Figure 1). Subsequent analyses of these cosmids and plasmids revealed two classes of genes that conferred an Acr^R^ phenotype; one resembled the previously identified *acuI*, but the other was wholly different.

### Metagenomic AcuI-like Medium Chain Dehydrogenase/reductases

Two cosmids (pBIO2079 and pBIO2081), and five pCR-XL-TOPO-based plasmids (pBIO2151, pBIO2152, pBIO2153, pBIO2154 and pBIO2155), each contained a gene whose product resembled AcuI. The locations of these *acuI*-like genes, in the context of flanking genes in the cloned metagenomic DNA, and the similarities of their gene products to polypeptides in known bacteria are shown in Tables S2, S3, S4, S5, S6, S7 and S8.

**Figure 2:**
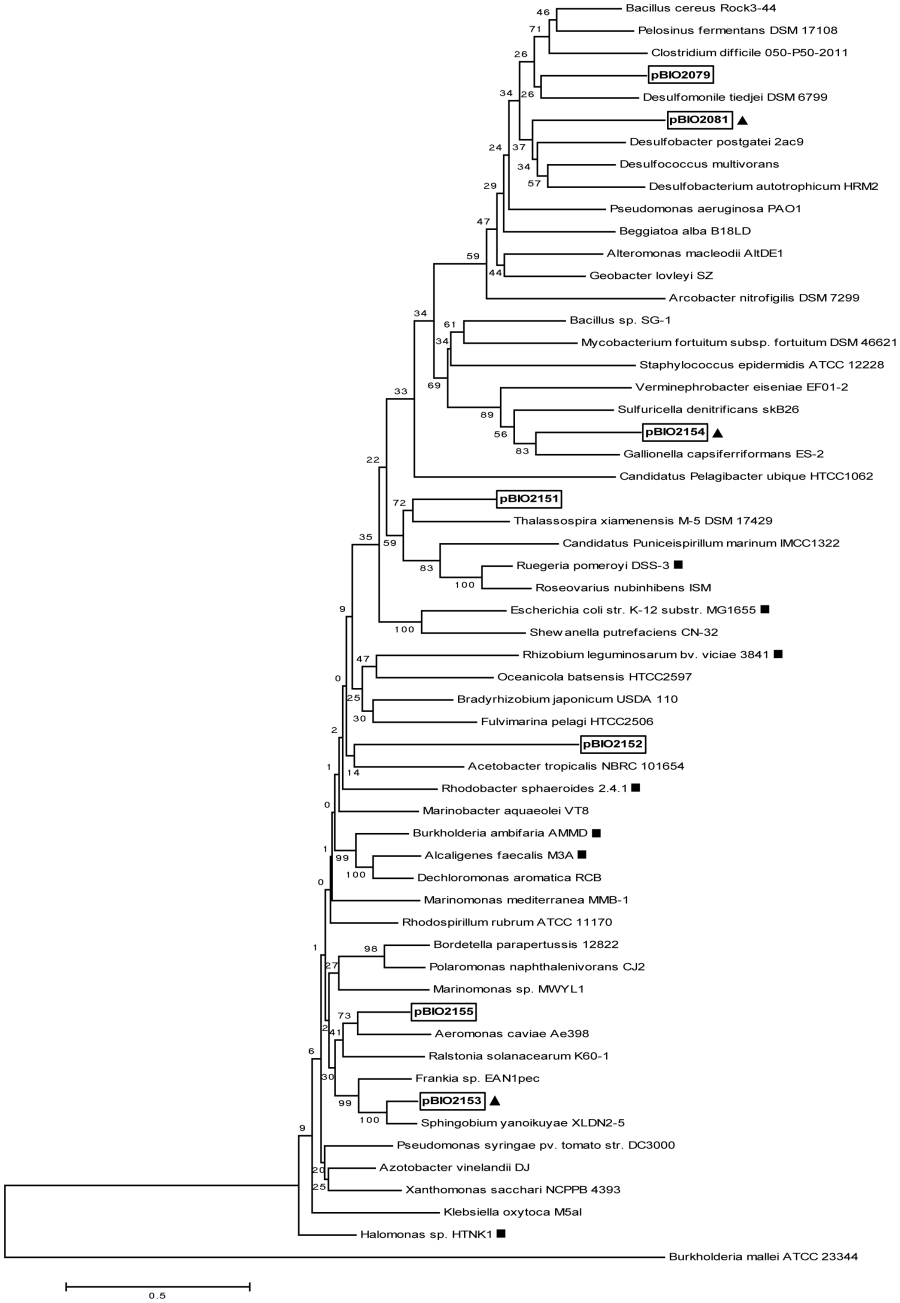
Phylogenetic tree of metagenomic AcuI-like proteins and other MDR012 subfamily proteins. In this neighbour-joining tree, the metagenomically identified AcuI enzymes are represented by the plasmid or cosmid from which they originate and are shown boxed, with those that also contain an adjacent CoA ligase marked with a black triangle. Other members of the MDR012 subfamily of the MDR superfamily in other bacteria are also included, with those that have been ratified as conferring an Acr^R^ being marked with black squares. The MDR039 subfamily polypeptide of *Burkholderia mallei* ATCC 23344 (BMAA0163, accession YP_104995) was used as the outlier sequence.

As illustrated in a neighbor-joining tree (Figure 2), the sequences of these seven AcuI-like metagenomic polypeptides were compared with each other and with ratified, AcuI polypeptides, and also with other AcuI homologues from a range of different bacterial taxa. Several of these newly obtained versions of AcuI closely resembled those in known, taxonomically diverse bacteria, most of which had no known links with acrylate and/or DMSP. This further demonstrates the diversity of the AcuI polypeptides and the lack of congruity between the amino acid sequences of the various AcuI gene products and the taxonomic status of the bacteria that harbour them. For example, the AcuI-like polypeptide of the Firmicute *Bacillus* sp. SG-1 more closely resembles that of the Actinomycete *Mycobacterium fortuitum* than that of another *Bacillus* species, *B. cereus* Rock3-44. Therefore, *acuI* has likely been subject to wide-range, repeated rounds of horizontal gene transfer.

Overall, these metagenomic screens considerably extend the diversity of functional AcuI-type enzymes that confer an Acr^R^ phenotype, so this may be a feature of all MDR012-type polypeptides, in diverse bacteria.

### Flanking Genes Next to *acuI* in Metagenomic Clones

For most of the individual metagenomic clones, all the sequenced genes within the cloned DNA are likely from the same general type of bacterium (Tables S2, S3, S4, S5, S6, S7 and S8), for example, Firmicutes (in pBIO2079), Sphingomonadales (pBIO2153) or betaproteobacteria (pBIO2154). Mostly, the predicted functions of the flanking genes differed in the various metagenomic cosmids and have no known link with acrylate. However, in pBIO2081, pBIO2153 and pBIO2154, *acuI* was next to a gene whose product was in the Pfam family PF00501 of AMP-binding long-chain fatty acid-CoA ligases; conceivably, these may add CoA to acrylate (or similar molecule) that, in turn is the substrate for the corresponding AcuI acrylyl-CoA reductase.

### A Metagenomically Derived Enoyl-acyl Carrier Protein (ACP) Reductase Confers an Acr^R^ Phenotype

The metagenomic screens also yielded pBIO2160, a pCR-XL-TOPO-based plasmid that conferred acrylate resistance (Figure 1), but in a very different way. The 5901 bps of insert DNA in pBIO2160, obtained from a cast-water biofilm, contains nine genes, none of which resembles *acuI* (Table S9). By sub-cloning individual genes, we identified one, termed *arkA* (cloned in pBIO2167), that was responsible for the Acr^R^ phenotype.

ArkA is a strongly predicted (e^−86^) Class II enoyl-[ACP] reductase C (Pfam family PF03060) with >70% identity to a gene product in many (though not all) genome-sequenced species of the genus *Bacillus* and its close relatives (Table S9). Furthermore, the products of the other eight genes in pBIO2160 also resembled those in these Firmicutes. We noted, though, that *arkA* is absent from some well-known species *of Bacillus* (*B. cereus, B. thuringiensis*, *B. anthracis*), some of which contain an *acuI-*like gene (see above).

To show that the *arkA-*like gene of a known species of *Bacillus* can confer an Acr^R^ phenotype, we amplified BMD_3924 of *B. megaterium* DSM 319, whose product is 61% identical to the metagenomic ArkA gene product of pBIO2160. When cloned into pET21a to form pBIO2195, BMD_3924 enhanced the acrylate resistance of the *E. coli* AcuI^−^ mutant J522 allowing growth at concentrations >1 mM.

**Figure 3:**
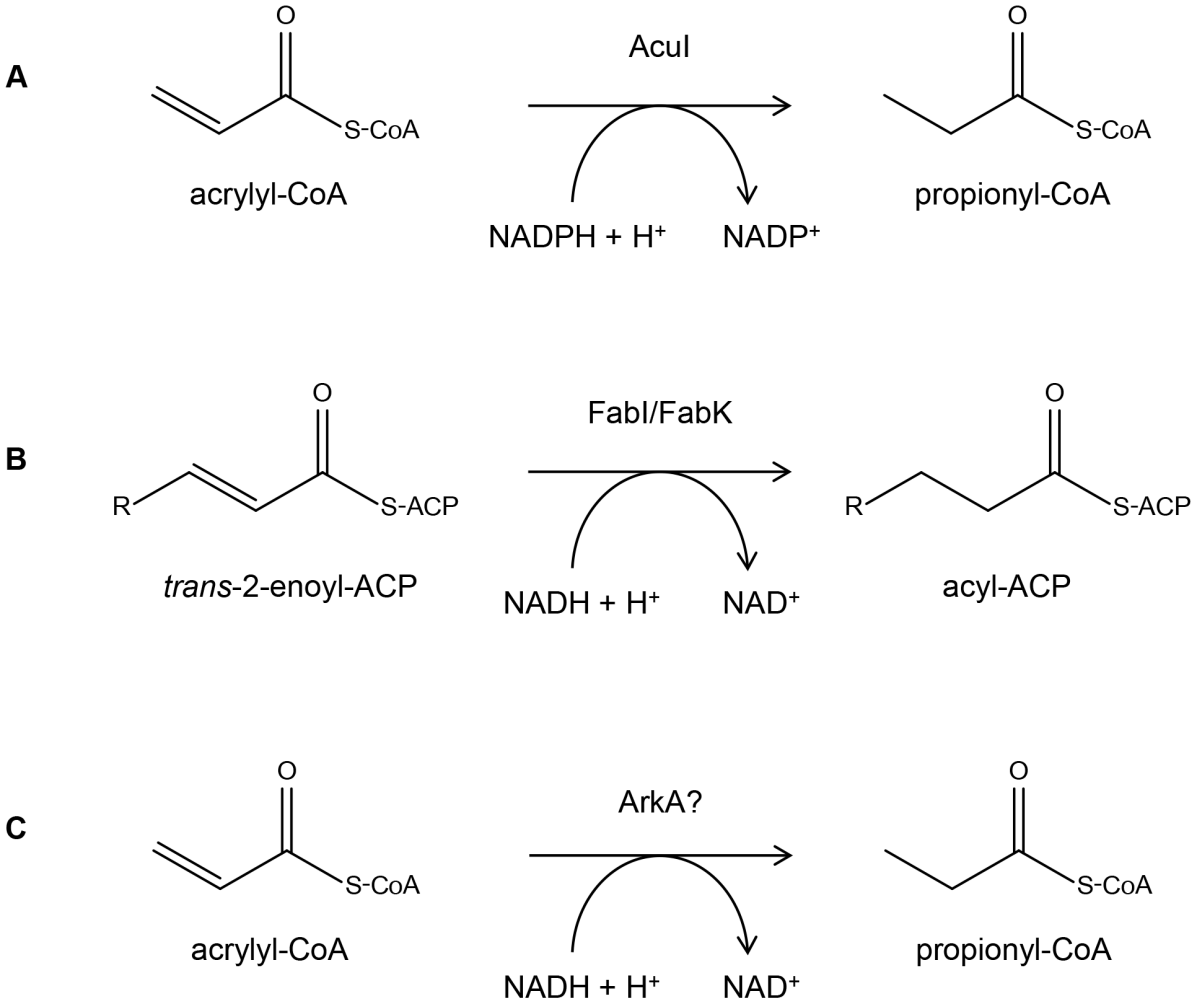
Predicted reactions for enzymes conferring an Acr^R^ phenotype and related fatty acid biosynthesis enzymes. Reaction A shows the conversion catalysed by AcuI [20]. Reaction B shows the conversion catalysed by FabI [40] and FabK [37]. Reaction C represents the ArkA-mediated conversion of acrylyl-CoA to propionyl-CoA predicted in this study.

The sequences of these Bacillaceae ArkA polypeptides placed them into a distinct cluster in Pfam family PF03060, which has two other groups, with only limited sequence identity (∼40%) to each other, and to the ArkA enzymes described here. One of these other groups is exemplified by a fungal 2-nitropropane dioxygenase, which removes the nitro group from various anionic nitroalkanes [35], [36]. Members of the other sub-group have a very different function; these correspond to FabK, first found in *Streptococcus* and the closely related *Enterococcus* [37]. In these bacteria, FabK catalyses the final step in fatty acid biosynthesis, reducing *trans*-2-enoyl-ACP to generate acyl-ACP (Figure 3). FabK was discovered because these two pathogens were completely resistant to triclosan [38], a widely used antimicrobial additive in various household products. Triclosan inhibits FabI [39], which occurs in many bacteria, including *E. coli*, and in eukaryotes, and which has the same biochemical function in fatty acid biosynthesis as FabK [40], [41], despite a lack of sequence similarity between these polypeptides (Figure 3).

Introduction of the cloned *fabK* gene of *Streptococcus* into *E. coli* confers high-level tolerance to triclosan, showing that FabK can functionally replace the triclosan-sensitive FabI of *E. coli* [37]. In contrast, when we introduced plasmids containing the metagenomic and *B*. *megaterium arkA* genes (in pBIO2167 and pBIO2195 respectively) into *E. coli*, there was no such effect; the two derivatives grew in 20 µM triclosan, the same maximal level as for *E. coli* with the empty vector.

Taken together, these observations show that most, but not all, strains of the Bacillaceae contain the enzyme ArkA. Although similar in sequence to FabK, the ArkA polypeptide is not involved in fatty acid biosynthesis, but may have some other, as yet unknown role. Nevertheless, the similarity of the ArkA and FabK sequences underpinned our naming of *arkA* (acrylate resistance FabK-type). In light of the reaction that is catalysed by *bona fide* FabK, it seemed likely that ArkA reduces acrylyl-CoA to propionyl-CoA when exogenous acrylate is present, the same bioconversion as that effected by AcuI (Figure 3).

### *Novosphingobium* Genes that Restore Acrylate Resistance to the *E. coli* AcuI^−^ mutant

Given the diversity of the types of genes that confer an Acr^R^ phenotype and the taxonomic and ecological range of bacteria that contain them, we took another approach to see if there were yet other ways in which bacteria can detoxify acrylate. This stemmed from our observation that the deduced proteomes of several genome-sequenced bacterial strains and genera lacked any of the known enzymes that act on acrylyl-CoA, namely AcuI and ArkA, and those in the Crenarchaea, *Chloroflexus aurantiacus* or *Clostridium propionicum* (see Introduction). One such genus is *Novosphingobium,* an alphaproteobacterium that is characterised by its ability to catabolise a wide range of organic aromatic compounds [42].

To identify any *Novosphingobium* gene(s) that conferred an Acr^R^ phenotype, we exploited a pre-existing genomic library of *Novosphingobium tardaugens* ARI-1, which comprised ∼23,000 cosmids, each with inserts ∼25 kb in size, cloned in pLAFR3. These plasmids were mobilised, *en masse,* into the *E. coli* AcuI^−^ mutant J523, selecting for transconjugants that grew on medium supplemented with 5 mM acrylate. One such colony was obtained and the cosmid, termed pBIO2170, that was responsible for conferring the Acr^R^ phenotype was studied in more detail.

**Figure 4:**
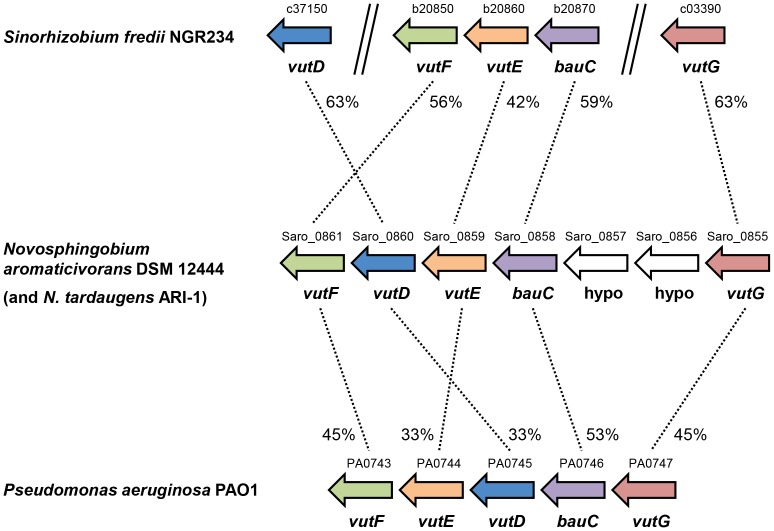
Gene maps of *Sinorhizobium, Novosphingobium* and *Pseudomonas bau*/*vut* genes. The arrangement of genes in *S. fredii* NGR234, *N. aromaticivorans* DSM 12444 and *Pseudomonas aeruginosa* PAO1 is shown, with the gene numbers above and gene names below. The gene arrangement in *N. tardaugens* ARI-1, in cosmid pBIO2170, is the same as that in the sequenced *N. aromaticivorans* strain. The % identity of the *N. aromaticivorans* gene products to the corresponding gene products in *Sinorhizobium* and *Pseudomonas* are shown by the dashed lines that link the homologous genes. Abbreviations: hypo = hypothetical protein.

**Figure 5:**
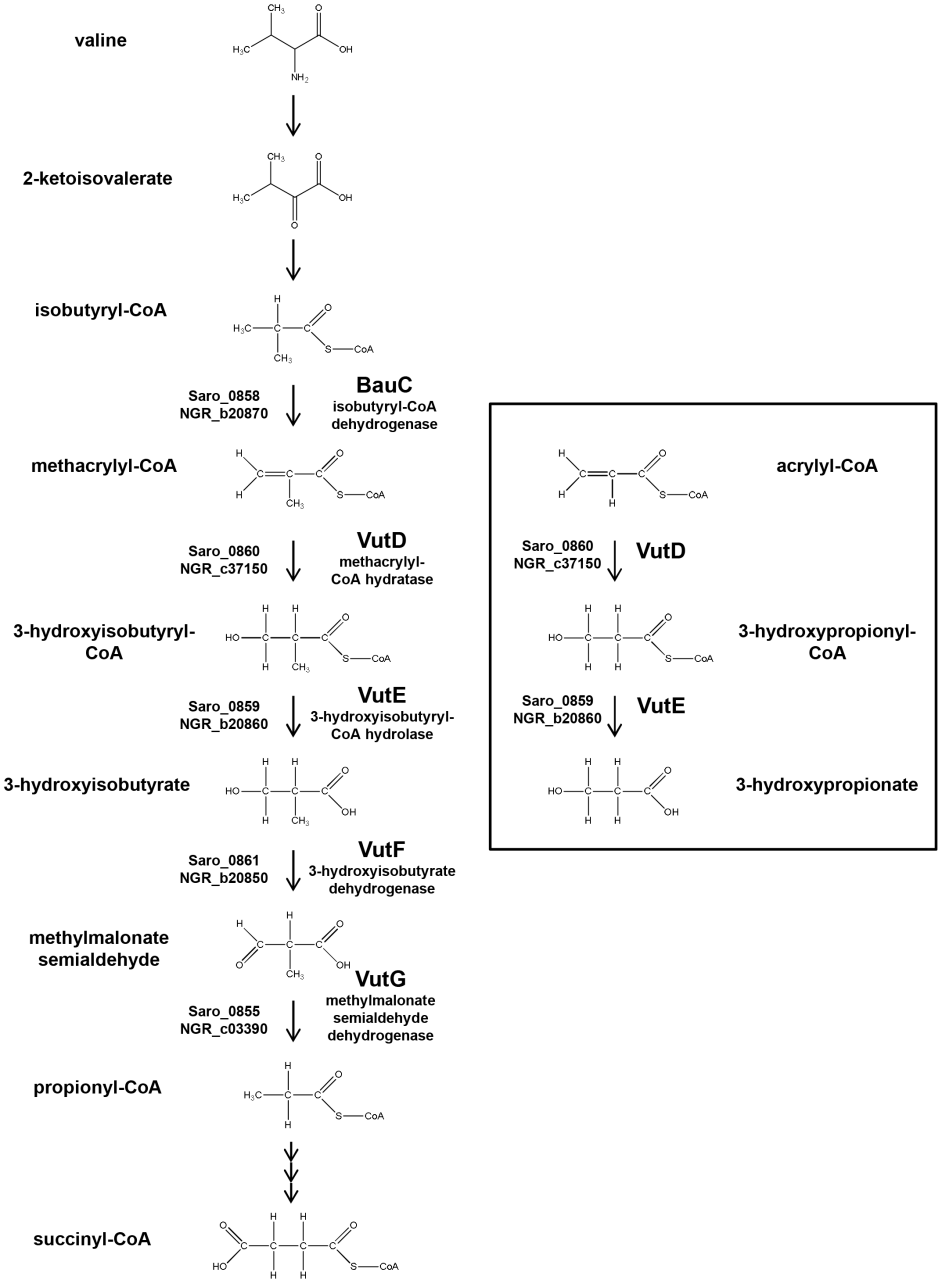
Valine oxidation pathway and predicted reactions in -CoA detoxification. The reactions and relevant enzymes of the valine oxidation pathway are shown, with the predicted reactions of acrylyl-CoA detoxification shown in the box. Reactions involving multiple steps are marked with multiple arrows. The gene numbers for genes in *N. aromaticivorans* and *S. fredii* that encode each enzyme are shown to the left of each reaction.

Sequencing the insert in pBIO2170 confirmed that the sequences and the synteny of the cloned genes corresponded closely to those in all the other five genome-sequenced *Novosphingobium* species, including the much-studied *N. aromaticivorans* DSM 12444 [43]. One predicted seven-gene operon, equivalent to Saro_0855-Saro_0861 in strain DSM 12444, (Figure 4 ) was of immediate interest, since it included five genes (*bauC, vutD, vutE, vutF* and *vutG* - see below) whose products are predicted to catalyse consecutive steps in the oxidative catabolism of valine. Significantly, an intermediate in this pathway is methacrylyl-CoA (MAC-CoA), which closely resembles acrylyl-CoA (Figure 5). The other two genes in the operon, corresponding to Saro_0856 and Saro_0857 in DSM 12444, encode short polypeptides of no known function, and with no homologues apart from in other *Novosphingobium* strains.

To confirm that gene(s) in this operon conferred an Acr^R^ phenotype, pBIO2170 was mutagenized with Tn*5lacZ* (see Materials and Methods) and the mutagenized cosmids were examined for any that no longer corrected the acrylate sensitivity of the *E. coli* AcuI^−^ mutant. All the insertions in these mutant cosmids were in the *vutD* or *vutE* genes, so-named because their products catalyse the corresponding steps in the valine utilisation pathway of *Pseudomonas putida* [44], [45].

Given the known toxicity of MAC-CoA [46] and its structural similarity to acrylyl-CoA, it was reasonable that the cloned *vutD* and *vutE* genes confer acrylate resistance by effecting the conversion of acrylyl-CoA to 3HP, via the intermediate 3HP-CoA. Figure 5 illustrates the *bona fide* valine catabolic pathway, as well as the likely alternative conversions that would occur when exogenous acrylate is supplied.

To test this and to study the roles of the bacterial *vutD* and *vutE* genes directly, we chose to work on another alphaproteobacterium, *Sinorhizobium fredii* NGR234, which is more readily grown and genetically amenable than *Novosphingobium*. Like *Novosphingobium*, *S. fredii* NGR234 lacks *acuI*, and we noted that it contains all the relevant *bau*/*vut* valine oxidation genes, although, unlike *Novosphingobium*, they are not contiguous. Thus, *bauC* (NGR_b20870), *vutE* (NGR_b20860) and *vutF* (NGR_b20850) are adjacent to each other on the large plasmid pNGR234b, but are unlinked to *vutD* (NGR_c37150) and *vutG* (NGR_c03390), which are both located (but separately from each other) on the *S. fredii* chromosome (Figure 4).

### The VutD and VutE Enzymes of *S. fredii* NGR234 Confer Acrylate Resistance

To examine directly if *vutD* (NGR_c37150) and *vutE* (NGR_b20860) of *S. fredii* NGR234 confer acrylate resistance, each of them was individually cloned into different, compatible vectors - pBluescript for *vutD* (forming pBIO2173) and pRK415 for *vutE* (forming pBIO2174) - and each of these two plasmids was used to transform the *E. coli* AcuI^−^ mutant J522. Whereas the cloned *vutE* gene had no effect on the mutant’s acrylate sensitivity, pBIO2173 (containing *vutD*) conferred partial resistance (tolerant to 5 mM acrylate). However, when a derivative of the *E. coli* mutant that contained *both* pBIO2173 *and* pBIO2174 was made, this was fully resistant, up to wild-type levels. Thus, it seems that the conversion of acrylyl-CoA to 3HP-CoA by VutD affords some protection, but that further resistance is conferred by VutE, via the subsequent conversion of 3HP-CoA to the less toxic 3HP [47].

### Effect of *Sinorhizobium fredii vutD* and *vutE* Mutations on Growth on Valine and Sensitivity to Acrylate

To confirm that VutD and VutE function in the valine oxidation pathway in *S. fredii* itself, we made insertional, genomic mutations in *vutD* (NGR_c37150) and *vutE* (NGR_b20860) of *S. fredii*, using pBIO1879, a Spc^R^-resistant derivative of the widely used suicide plasmid pK19*mob* (see Materials and Methods). One ratified mutant for each of the genes, designated strains J554 (VutD^−^) and J555 (VutE^−^), was compared with the wild type, as follows. Whereas wild type *S. fredii* NGR234 grew well on *L-*valine (3 mM) as sole carbon source, to a final OD_600_ of ∼0.3, neither the VutD^−^ nor the VutE^−^ mutants showed any signs of growth on this amino acid, although both grew normally on succinate, to an OD_600_ of 0.91 and 0.88 respectively.

**Figure 6:**
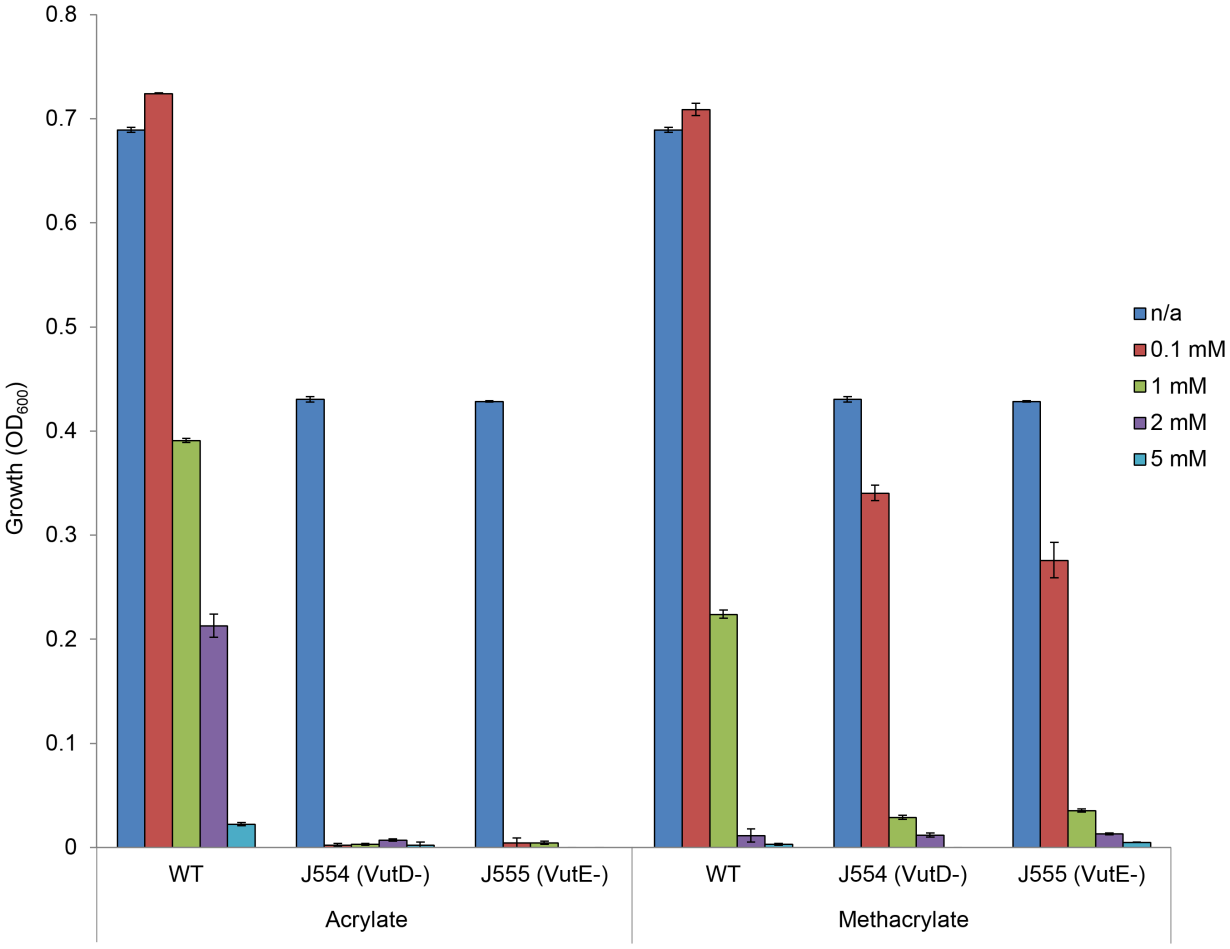
Growth of *S. fredii* NGR234 wild type, VutD^−^ and VutE^−^ mutants with acrylate or methacrylate. Cultures of *S. fredii* wild type (WT), and VutD^−^ (J554) and VutE^−^ (J555) mutant strains were inoculated into RM minimal medium containing succinate as carbon source and increasing concentrations of either acrylate or methacrylate, and incubated at 28°C for 24 hours. Growth was recorded as optical density at a wavelength of 600 nm (OD_600_). Experiments were repeated and error bars showing standard deviations based on three replicates are shown. Concentrations of acrylate or methacrylate used are shown in the colour key (n/a = no addition).

As predicted, the *S. fredii* VutD^−^ and VutE^−^ mutants were more sensitive to acrylate and to methacrylate than the wild type, which grew in the presence of either compound at concentrations of 1 mM; in contrast, the VutD^−^ and VutE^−^ mutants (J554 and J555) showed no growth at 0.1 mM (Figure 6).

Taken together, these results confirm that the VutD and VutE enzymes of *S. fredii* are required for valine oxidation, and that mutations in either of these genes also affect the ability of *S. fredii* to tolerate the toxic effects of acrylate or methacrylate.

### Relationship of the *VutD* and *VutE* Enzymes to the Previously Identified AcuK Acrylyl-CoA Hydratase

VutD is in the same super-family (Pfam PF00378) of enoyl-CoA hydratase/isomerases as AcuK, an enzyme described in two bacteria that catabolise DMSP, namely *Halomonas* sp. HTNK1 [12] and *Alcaligenes faecalis* M3A [13], in both of which *acuK* is clustered with the *ddd* genes required for DMSP catabolism. The ability of these two species to grow on acrylate (via DMSP cleavage) requires AcuK, which is predicted to convert acrylyl-CoA to 3HP-CoA, the same as that described for VutD (see above). Although we noted that VutD does not lie within the sub-group of PF00378 polypeptides that includes ratified AcuK enzymes, VutD and AcuK are similar in their sequences (ca 60% identical). AcuK (in *Halomonas*) and VutD (in *S. fredii*) also show similarity to the acrylyl-CoA hydratase (SPO0147) identified in the DMSP catabolic pathway of *R. pomeroyi* [48], being 55% and 63% identical respectively to the sequence of this enzyme, which has been shown to convert acrylyl-CoA to 3HP-CoA. These observations prompted us to test if, like VutD, AcuK can confer resistance to acrylate, as follows. A plasmid, pBIO1729, containing *acuK* of *Halomonas* sp. HTNK1 [12] was introduced into the *E. coli* AcuI^−^ mutant strain J522. The resultant transformant displayed a slight increase in acrylate tolerance, similar to that seen (above) with the cloned *vutD*. However, when the cloned *acuK* gene was introduced into the *E. coli* mutant that already harboured *vutE* (in plasmid pBIO2174), this fully restored acrylate resistance (data not shown). This was analogous to what was seen, above, with the synergistic effects of *vutD* plus *vutE*.

Interestingly, the *ddd*/*acu* gene clusters of both *Alcaligenes faecalis* M3A and *Halomonas* sp. HTNK1 also include an *acuI* gene, as well as *acuK* (see above), so they may have two separate protective systems against acrylate-mediated toxicity. The reason for this is unclear but one explanation could be that AcuI is a dedicated detoxification system for acrylyl-CoA, whereas AcuK is mainly used to catabolise acrylate as a source of carbon, but the way(s) in which such functional compartmentalisation may occur are not clear.

## Discussion

Almost by definition, functional metagenomics, as first envisaged by Jo Handelsman, provides a powerful method to isolate “novel” genes that would not have been recognised on the basis of scanning the sequences of metagenomes (or genomes). This approach has been most widely aimed at isolating novel genes that confer functions of biotechnological relevance (e.g. cellulases or lipases [49], [50]). But, as shown here, it can also uncover unexpected genes that may be involved in more fundamental metabolic function(s), as well as extending the diversity of previously identified genes that confer the function under study (*acuI* in this case).

The most frequently encountered metagenomic genes that corrected the acrylate sensitivity of the *E. coli* AcuI^−^ mutant were themselves related to *acuI.* These varied considerably in their sequences, and likely came from the genomes of a wide range of bacterial taxa. Nevertheless, all these seven AcuI polypeptides were squarely placed within the medium chain reductase/dehydrogenase MDR012 sub-family [23], as had been all the previously ratified AcuI polypeptides that confer an Acr^R^ phenotype [22]. It remains to be seen if *all* MDR012 polypeptides confer acrylate resistance, but, judging by the MDR012 sequence space that is occupied by those for which this has been confirmed (Figure 2), and the taxonomic range of the bacteria in which these occur, it would be surprising if this were not the case.

For those bacteria that catabolise DMSP, generating large amounts of acrylate and its even more toxic CoA-ligated derivative, the presence of an enzyme that converts acrylyl-CoA to a less noxious product makes good sense, especially if the corresponding gene is closely linked to the *ddd* genes that are responsible for making the acrylate and if the expression of that gene is markedly enhanced by the acrylate co-inducer. And this is exactly what occurs in several taxonomically diverse bacteria, in which their *acuI* is clustered with the *ddd* or *dmdA* genes [3], [22]. But what of those many other bacteria, which on the face of it, do not encounter acrylate? As a caveat to that last query, we would say that although we do not normally associate acrylate as part of the chemical milieu of many bacteria (notably *E. coli*), this may not necessarily be true. Many marine animals (from krill to penguins to seals) *do* eat DMSP-containing vegetation either directly or indirectly, and, indeed, seaweeds contribute to the human diet in many parts of the world. Acrylate was shown to affect the gut microflora of penguins [10], and DMSP-catabolising bacteria were readily obtained from guts of herrings [51]. So, enteric bacteria, at least, might encounter acrylate, at a global level, sufficiently often to maintain a selective advantage on those cells that can detoxify it.

However, if exogenous acrylate does *not* provide the selection pressure to maintain *acuI,* two possible scenarios must be considered. The first is that acrylyl-CoA is generated endogenously, even though the presence of this molecule has only been documented in a few cases over and above those bacteria that catabolise DMSP such as those cases, described above, in *Chloroflexus, Clostridium* and some Archaea. It has also been predicted that acrylyl-CoA is formed from lactoyl-CoA in lactate metabolism [52]–[54], propionyl-CoA in propionate metabolism [55] and the β-oxidation pathway of glucose fermentation [56], and β-alanyl-CoA in β-alanine metabolism [57], but these suggestions have not been directly verified.

A second general explanation is that acrylyl-CoA may not be the *bona fide* “natural” substrate. Certainly, we are unaware of any report that describes acrylyl-CoA in *E. coli* cells, despite the very strong affinity of the AcuI (YhdH) enzyme for this molecule in this species [21]. A more detailed study of the *acuI* (*yhdH*) mutants of *E. coli* might shed light on the “proper” role of these genes. To date, their only recognised phenotype is their marked sensitivity to acrylate, at concentrations as low as 20 µM, some two orders of magnitude less than in the wild type [22]. A more wide-ranging and subtle search of any other phenotypic changes, including those that may alter the metabolome, and performed under various environmental conditions might reveal the *bona fide* pathway that involves AcuI in these bacteria. Of course, such an approach could also be applied to other, genetically amenable bacteria that contain AcuI-like enzymes. Given the relatively wide diversity of the amino acid sequences in these different AcuI-type enzymes, the “correct” substrate/pathway need not necessarily be the same in all bacteria, even though they can all recognise acrylyl-CoA. In connection with this, it is noteworthy that in three metagenomic clones, in which the cloned DNA was likely derived from genomes of different delta-, alpha- and gammaproteobacteria, their *acuI* gene was immediately 5′ of a gene that likely encoded a CoA-ligase. Furthermore, we also noted close linkage of *acuI-*like genes and a gene that encodes a CoA-ligase in the genomes of several known bacteria (e.g. the betaproteobacteria *Azoarcus* sp. KH32C and *Dechloromonas aromatica* RCB, and the Actinomycete *Frankia*). Although in the same polypeptide family, these various CoA-ligases are not very closely related to each other (<30% identical). However, the partial amino acid sequence of the ligase from pBIO2154 showed 47% identity (over ∼500 amino acids of its N-terminus) to the only known (so far) acrylyl-CoA ligase [48]. In these cases, the products of these cloned genes may ligate CoA to an acrylate-like molecule, prior to its reduction by the product of the neighbouring *acuI-*like gene. However, the diversity of these ligases, together with the known low specificity of enzymes of this type [58], again preclude any confident predictions on what the natural substrate might be; again, an analysis of the corresponding mutants may be needed.

As already noted, many bacterial strains, genera and even whole phyla lack an *acuI-*like gene and this had underpinned our use of functional metagenomics to establish if this patchy distribution might be due to the role of AcuI being undertaken by other enzymatic systems. In terms of proof of concept, this was successful. Despite its lack of sequence similarity to AcuI, the *arkA* gene conferred an Acr^R^ phenotype and likely catalyses the same overall reaction, namely the reduction of acrylyl-CoA to its propionyl derivative (Figure 3), though by a different molecular mechanism, in which NAD is the predicted coenzyme, in contrast to the NADP that occurs in AcuI [59].

The same questions that were raised with regard to the “real” function of *acuI* in (for example) *E. coli* also apply to the strains of *Bacillus* that harbour *arkA.* We do not know if the *bona fide* substrate is acrylyl-CoA or the pathway in which the ArkA enzyme participates. Whatever its function, it does not seem to be involved in fatty acid synthesis, despite the significant sequence similarity of ArkA and FabK, since *arkA* of *B. megaterium* did not rescue *E. coli* from its sensitivity to triclosan. Also, several strains of *Bacillus* that contain *arkA*, including *B. megaterium,* contain *fabL,* which encodes the *bona fide* enzyme that catalyses the final step in fatty acid biosynthesis in many strains of *Bacillus* [60]. As discussed above for *acuI,* the isolation and characterisation of an ArkA^−^ mutant in a genetically amenable *Bacillus* strain (e.g. *B. subtilis*) might provide useful insights, but this has not yet been done.

Close relatives of the metagenomically obtained *arkA* (and its neighbouring genes) are restricted to some strains of *Bacillus* and very closely related genera of Firmicutes. But, as with *acuI,* the distribution of *arkA* is sporadic, being absent from several *Bacillus* species, including the well-known *B. cereus, B. anthracis* and *B. thuringiensis.* Do these strains not need an ArkA-type enzyme activity - or can it be substituted by yet another enzyme?

With the discovery of the *vutD* and *vutE* genes of *Novosphingobium* and *Sinorhizobium,* our complementary approach, in which we mined the genomes of bacteria that lack *arkA* or *acuI* for genes that conferred an Acr^R^ phenotype, also proved successful. In this case, though, unlike that for *arkA,* pre-existing knowledge of the functions of these genes provided a ready explanation for the Acr^R^ phenotype. Thus, the conversion, in two steps of the valine oxidation pathway, of the highly toxic [46] MAC-CoA to 3-hydroxyisobutyryl-CoA and then to 3-hydroxyisobutytrate would correspond to the generation of 3-hydoxypropionate if cells were supplied with exogenous acrylate (Figure 5). Our finding that maximal protection against the effects of acrylate requires *both vutD and vutE* provides strong evidence that a CoA-ligated intermediate (in this case, the VutE substrate, 3-hydroxypropionyl-CoA) is significantly more cytotoxic than the unmodified 3HP itself, but that both are less harmful than the acrylyl-CoA.

The valine oxidative pathway is widespread, in many bacteria, animals and plants [46], [61], [62]. The repercussions of the toxic intermediate MAC-CoA may even underpin an unusual feature of this pathway (Figure 5), in which an activated acyl group (in 3-hydroxyisobutyryl-CoA) is destroyed, only for another such molecule (propionyl-CoA) to be made later as a means of rapidly removing the potentially harmful MAC-CoA [46]. Certainly, genetic defects in this pathway cause serious pathologies in humans [63] and in plants [64].

Despite the extension of the different types of enzyme that can confer an Acr^R^ phenotype, there are still many bacteria that contain none of the polypeptides that resemble AcuI, ArkA, or VutDE described above, or the AcrABC enzyme that converts acrylyl-CoA to propionyl CoA in the Clostridia [30]. These may simply have no need for such an enzymatic activity but, if these have yet other ways of dealing with acrylate, it should be relatively straightforward to identify these by making fresh genomic libraries from such ‘null’ bacteria and to screen these, in turn, for any cloned, functional genes that confer an Acr^R^ phenotype.

Similarly, more wide-ranging screenings of metagenomic libraries that sample a wider range of environments (Including marine ones) may uncover yet other types of genes that confer an Acr^R^ phenotype. Although the ‘natural’ functions of any such genes may not be forthcoming - as in the case of *arkA* - this approach could nonetheless point to the *general* functions of at least a few of the massive numbers of genes of unknown function that crowd the databases of genomes and metagenomes.

## Materials and Methods

### Strains, Plasmids and Growth Conditions

Table S10 shows the bacterial strains and plasmids. *E. coli* was grown on LB media [65] at 37°C. *Sinorhizobium fredii* NGR234 [66] and *Rhizobium leguminosarum* 3841 [67] grew on TY complete or *Rhizobium* minimal (RM) medium (SY medium [68] modified with 3 g L^−1^Tris) at 28°C. *Novosphingobium tardaugens* ARI-1 [69] was grown on nutrient broth (5 g L^−1^ peptone, 3 g L^−1^ meat extract, pH7) at 28°C. Antibiotics were used at these concentrations (µg ml^−1^): rifampicin (rif; 20), kanamycin (kan; 20 for *E. coli*; 200 for both *S. fredii* and *R. leguminosarum*), streptomycin (str; 400), spectinomycin (spc; 50 for *E. coli;* 200* for S. fredii*) and tetracycline (tet; 5).

To test growth of *S. fredii* NGR234 on different carbon sources, late log phase cells were washed, diluted 1/100 into 5 ml RM containing the appropriate carbon source, then incubated with shaking at 28°C; growth was monitored at OD_600_. Sensitivity of *E. coli* to acrylate was tested by placing 10 µl aliquots of various dilutions of freshly grown cells onto LB agar plates containing appropriate concentrations of acrylate or other test compounds, then incubated for 16 hours. *S. fredii* cultures were incubated for 48 hours in 5 ml TY, then diluted 1/100 into 5 ml RM medium containing 10 mM succinate as carbon source, plus different concentrations of the test compounds. After 24 hours shaking incubation, the OD_600_ was determined.

### *In vivo* and *in vitro* Genetic Manipulations

Transfer of plasmids by conjugation, using helper plasmid pRK2013 [70], and transformation of *E. coli,* were done as in Wexler *et al*. [71] and by electroporation, essentially as in Hanahan [72]. *E. coli* strain 803 [73] was the usual host for transformation of most plasmids; JM101 [74] was used as the host for pBluescript. PCR primers containing appropriate restriction sites are shown in Table S11.

### Construction of Mutant Strains

An insertional mutation into *acuI* (*yhdH*) of wild type *E. coli* J521 was made using the lambda red recombinase system [75]. Primers designed to amplify the Spc^R^ cassette from plasmid pHP45Ω [76] also contained 40 bp flanking regions, corresponding to sequences immediately 5′ and 3′ of *yhdH* (Table S11). The resulting PCR product was electroporated into *E. coli* J521 carrying a lambda red recombinase expression plasmid, then plated on LB Spc plates and incubated at 30°C. One Spc^R^ colony, termed strain J557, was picked and the insertional mutation in *yhdH* was confirmed by PCR, and by the mutant’s acrylate-sensitive phenotype.

Insertional mutations in the *S. fredii* NGR234 genome were made using the pK19*mob* suicide vector system [77]. Fragments internal to the *S. fredii* genes NGR_c37150 and NGR_b20860 were each amplified and cloned into pBIO1879 [78], a Spc^R^ derivative of pK19*mob*, to form plasmids pBIO2176 and pBIO2177 respectively. These plasmids were each transferred to *S. fredii* NGR234 by triparental conjugations. Single crossover events, in which the incoming plasmid had inserted into the corresponding target genes, were selected on TY plates containing Str (to kill *E. coli* donor strains), Spc and Kan (resistances conferred by pBIO1879). Strains with verified mutations in the genes NGR_c37150 (*vutD*) and NGR_b20860 (*vutE*) were termed J554 and J555 respectively.

The pLAFR3-based cosmid pBIO2170, which contains cloned *N. tardaugens* ARI-1 genomic DNA, including the *bau*/*vut* gene cluster, was randomly mutated using transposon Tn*5lacZ*. Plasmid pBIO2170 DNA was transformed into *E. coli* strain A118, which has a chromosomal copy of Tn*5lacZ* [79]. One transformant was then used as the donor in a triparental conjugational mating with *R. leguminosarum* bv. *viciae* 3841 as the recipient, selecting for *Rhizobium* transconjugants that harboured pBIO2170::Tn*5lacZ* on Str (*Rhizobium*), Kan (Tn*5lacZ*) and Tet (pLAFR3 cosmid). Cosmid DNA was prepared from ca. 200 transconjugants and used to transform *E. coli* strain 803, an efficient host for transformation with large plasmid/cosmids. Approximately 100 individual *E. coli* transformants were individually used as donors in triparental matings to the *E. coli* Rif^R^ AcuI^−^ mutant strain J523. Any mutant cosmids that no longer corrected the acrylate sensitivity of the J523 mutant were studied further.

### Library Construction

The vector for the genomic library of *Novosphingobium tardaugens* ARI-1 was the wide host-range, mobilisable, cosmid pLAFR3 [80], which can accommodate inserts of ∼25 kb. These libraries were made essentially as described by Curson *et al*. [19].

Metagenomic libraries [34] from a wastewater treatment plant (WWTP) in Whitlingham, Norfolk, UK, also used pLAFR3 as the vector and metagenomic small-insert libraries in pCR-XL-TOPO were made as in Nacke *et al*. [81]. Details of the libraries and their construction are shown in Table S1.

### DNA Sequencing

End-sequencing or whole sequencing of inserts in plasmids and cosmids was done by the Göttingen Genomics Laboratory, Göttingen, Germany or by Genome Enterprise Ltd, TGAC, Norwich, UK. DNA sequence data was submitted to NCBI with the accession numbers KJ531199 (pBIO2079), KJ531206 (pBIO2081), KJ531200 (pBIO2151), KJ531205 (pBIO2152), KJ531204 (pBIO2153), KJ531203 (pBIO2154), KJ531202 (pBIO2155), KJ531201 (pBIO2160).

### Bioinformatics

Sequences were compared to the NCBI sequence databases using BLASTX and BLASTP [82]. Protein alignments and neighbour-joining phylogenetic trees employed MEGA v5.10 [83] with 100 bootstrap replications.

## Supporting Information

Table S1 **Library information.**
(DOCX)

Table S2 **Details of cosmid pBIO2079.**
(DOCX)

Table S3 **Details of cosmid pBIO2081.**
(DOCX)

Table S4 **Details of plasmid pBIO2151.**
(DOCX)

Table S5 **Details of plasmid pBIO2152.**
(DOCX)

Table S6 **Details of plasmid pBIO2153.**
(DOCX)

Table S7 **Details of plasmid pBIO2154.**
(DOCX)

Table S8 **Details of plasmid pBIO2155.**
(DOCX)

Table S9 **Details of plasmid pBIO2160.**
(DOCX)

Table S10 **Strains and plasmids used in this study.**
(DOCX)

Table S11 **Oligonucleotide primers used in this study.**
(DOCX)
