# Supplementary material for: Screening of Metagenomic and Genomic Libraries Reveals Three Classes of Bacterial Enzymes That Overcome the Toxicity of Acrylate
Source: PLoS One. 2014 May 21;9(5):e97660. doi: 10.1371/journal.pone.0097660 (PMC4029986; doi:10.1371/journal.pone.0097660)
Supplement: Table S1 — Library information. (DOCX) [file pone.0097660.s001.docx]

**Table S1. Library information.**

| **Library** | **Source** | **Vector and enzyme/cloning method** | **No. of clones** | **Reference** |
| --- | --- | --- | --- | --- |
| GLI | Primary sedimentation tank, WWTP, Norfolk, UK | pLAFR3, *Bam*HI (insert DNA cut with *Sau*3A) | ~11,000 | Wexler *et al*. (2005) |
| GLII | Activated sludge, WWTP, Norfolk, UK | pLAFR3, *Bam*HI (insert DNA cut with *Sau*3A) | ~80,000 | Wexler *et al*. (2005) |
| GLIII | Anaerobic digestor, WWTP, Norfolk, UK | pLAFR3, *Bam*HI (insert DNA cut with *Sau*3A) | ~110,000 | Wexler *et al*. (2005) |
| WB5.4 | Cast water biofilm, Westerhöfer Bach, Germany | pCR-XL-TOPO (TOPO cloning, Invitrogen) | ~707,000 | This work |
| E2 | Fly compost, Bioenergiezentrum Göttingen, Germany | pCR-XL-TOPO (TOPO cloning, Invitrogen) | ~36,000 | This work |
| E3 | Fly compost, Bioenergiezentrum Göttingen, Germany | pCR-XL-TOPO (TOPO cloning, Invitrogen) | ~530,000 | This work |
| E4 | Fly compost, Bioenergiezentrum Göttingen, Germany | pCR-XL-TOPO (TOPO cloning, Invitrogen) | ~28,000 | This work |
| *Novosphingobium tardaugens* ARI-1 | *N. tardaugens* ARI-1 genomic DNA | pLAFR3, *Eco*RI | ~23,000 | This work |
| *Sinorhizobium fredii* NGR234 | *S. fredii* NGR234 genomic DNA | pLAFR3, *Eco*RI | ~22,000 | This work |

Reference:

Wexler M, Bond PL, Richardson DJ, Johnston AW (2005) A wide host-range metagenomic library from a waste water treatment plant yields a novel alcohol/aldehyde dehydrogenase. Env Microbiol 7: 1917-1926.
