# Supplementary material for: Screening of Metagenomic and Genomic Libraries Reveals Three Classes of Bacterial Enzymes That Overcome the Toxicity of Acrylate
Source: PLoS One. 2014 May 21;9(5):e97660. doi: 10.1371/journal.pone.0097660 (PMC4029986; doi:10.1371/journal.pone.0097660)
Supplement: Table S2 — Details of cosmid pBIO2079. (DOCX) [file pone.0097660.s002.docx]

**Table S2. Details of cosmid pBIO2079**. Source: activated sludge, Whitlingham wastewater treatment plant, UK.

| **Gene (a)** | **Protein type of closest homologue (b)** | **Bacterial species of closest homologue (c)** | **Taxonomic status (d)** | **Gene ID/locus tag (e)** | **E value (f)** |
| --- | --- | --- | --- | --- | --- |
| A | hypothetical protein (transposase, IS4 family) | *Syntrophomonas wolfei* subsp. wolfei str. Göttingen | Firmicutes; Clostridia; | Swol_0138 | 0.0 |
| B | hypothetical protein | no significant homologue | - | - | - |
| C | TetR family transcriptional regulator | *Syntrophomonas wolfei* subsp. wolfei str. Göttingen | Firmicutes; Clostridia; | Swol_2450 | 1e^-49^ |
| D | ABC transporter substrate-binding protein | *Syntrophomonas wolfei*  subsp. wolfei str. Göttingen | Firmicutes; Clostridia; | Swol_1119 | 3e^-120^ |
| E | PAS/PAC sensor-containing diguanylate cyclase | *Bacillus cellulosilyticus* DSM 2522 | Firmicutes; Bacilli; | Bcell_2358 | 1e^-67^ |
| F | AcuI-like | *Pelosinus fermentans* DSM 17108 | Firmicutes; Negativicutes; | FR7_1103 | 9e^-115^ |
| G | uridylate kinase | *Syntrophomonas wolfei* subsp. wolfei str. Göttingen | Firmicutes; Clostridia; | Swol_2454 | 1e^-150^ |
| H | hypothetical protein (transposase, IS4 family) | *Syntrophomonas wolfei* subsp.  wolfei str. Göttingen | Firmicutes; Clostridia; | Swol_0138 | 0.0 |

A

B

G

F

E

C

D

H

The features of the genes are shown in tabular and diagrammatic forms. In the table, the gene letter in Column (a) corresponds to that in the figure below. Column (b) shows the predicted general function of the gene product, the species {column (c)} and taxonomic status {column(d)} of the bacterium that harbours the closest homologue, whose gene tag is shown in column (e) and whose BLASTP E value in comparison to the metagenomic polypeptide is in column (f). The blue row indicates the individual genes/proteins that confer acrylate resistance. In the figure, arrows indicate locations of genes in the cloned DNA, with the gene that confers acrylate resistance being in black.
