# Supplementary material for: Screening of Metagenomic and Genomic Libraries Reveals Three Classes of Bacterial Enzymes That Overcome the Toxicity of Acrylate
Source: PLoS One. 2014 May 21;9(5):e97660. doi: 10.1371/journal.pone.0097660 (PMC4029986; doi:10.1371/journal.pone.0097660)
Supplement: Table S4 — Details of plasmid pBIO2151. (DOCX) [file pone.0097660.s004.docx]

**Table S4. Details of plasmid pBIO2151.** Source: cast water biofilm, Westerhöfer Bach, Germany.

| **Gene** | **Protein type of closest homologue** | **Bacterial species of closest homologue** | **Taxonomic status** | **Gene ID/locus tag** | **E value** |
| --- | --- | --- | --- | --- | --- |
| A | hypothetical protein | *Methylobacterium nodulans* ORS 2060 | α-proteobacteria; Rhizobiales; | Mnod_3072 | 9e^-36^ |
| B | AcuI-like | *Thalassospira xiamenensis* M-5 | α-proteobacteria; Rhodospirillales; | TH3_21038 | 5e^-113^ |
| C | hypothetical protein | α-proteobacterium BAL199 | α-proteobacteria; | BAL199_07333 | 4e^-54^ |
| D | N-acetylmuramoyl-L-alanine amidase | *Magnetospirillum* sp. SO-1 | α-proteobacteria; Rhodospirillales; | H261_21813 | 4e^-85^ |
| E | Metal-dependent hydrolase of the beta-lactamase superfamily I | *Caenispirillum salinarum* AK4 | α-proteobacteria; Rhodospirillales; | C882_3868 | 1e^-93^ |

E

D

A

C

B

The features of the genes are shown in tabular and diagrammatic forms. In the table, the gene letter in Column (a) corresponds to that in the figure below. Column (b) shows the predicted general function of the gene product, the species {column (c)} and taxonomic status {column(d)} of the bacterium that harbours the closest homologue, whose gene tag is shown in column (e) and whose BLASTP E value in comparison to the metagenomic polypeptide is in column (f). The blue row indicates the individual genes/proteins that confer acrylate resistance. In the figure, arrows indicate locations of genes in the cloned DNA, with the gene that confers acrylate resistance being in black.
