# Supplementary material for: Screening of Metagenomic and Genomic Libraries Reveals Three Classes of Bacterial Enzymes That Overcome the Toxicity of Acrylate
Source: PLoS One. 2014 May 21;9(5):e97660. doi: 10.1371/journal.pone.0097660 (PMC4029986; doi:10.1371/journal.pone.0097660)
Supplement: Table S10 — Strains and plasmids used in this study. (DOCX) [file pone.0097660.s010.docx]

**Table S10. Strains and plasmids used in this study.**

| **Bacterial strains** | **Characteristics** | **Reference** |
| --- | --- | --- |
| *Escherichia coli* 803 | *E. coli* host used for transformation of large plasmids, Met^-^ | Wood (1966) |
| JM101 | *E. coli* host used for transformation of pBluescript and blue-white screening, Thi^-^ | Yanisch-Perron *et al*. (1985) |
| A118 | *E. coli* strain containing chromosomal Tn*5lacZ* insertion, used for transposon mutagenesis; kan^r^ | Simon *et al*. (1989) |
| J521 | *E. coli* wild type strain BW25113 | Baba *et al*. (2006) |
| J522 | KEIO collection mutant in *acuI* of *E. coli* BW25113; kan^r^ | Baba *et al*. (2006) |
| J523 | Rifampicin-resistant derivative of *E. coli* strain J522; rif^r^ | This work |
| J557 | Spectinomycin-resistant *acuI* mutant derivative of *E. coli* strain J521; spc^r^ | This work |
| *Rhizobium leguminosarum* bv. viciae 3841 | Wild type strain; str^r^ | Young *et al*. (2006) |
| *Novosphingobium tardaugens*  ARI-1 | Wild type strain | Fujii *et al*. (2003) |
| *Sinorhizobium fredii* NGR234 | Wild type strain; str^r^ | Trinick (1980) |
| J554 | *S. fredii* NGR234with a mutation in NGR_c37150 (*vutD*); str^r^, kan^r^, spc^r^ | This work |
| J555 | *S. fredii* NGR234with a mutation in NGR_b20860 (*vutE*); str^r^, kan^r^, spc^r^ | This work |
| **Plasmids** |  |  |
| pLAFR3 | Wide host-range cosmid vector used genomic and large insert metagenomic libraries; tet^r^ | Staskawicz *et al.* (1987) |
| pRK2013 | Mobilising plasmid in tri-parental crosses; kan^r^ | Figurski and Helinski (1979) |
| pET21a | Used for expression of cloned genes in *E. coli*; amp^r^ | Novagen |
| pBluescript SK- | Used for expression of cloned genes in *E. coli*; amp^r^ | Agilent |
| pRK415 | Used for expression of cloned genes in *E. coli*; tet^r^ | Keen *et al*. (1988) |
| pCR-XL-TOPO | TA cloning vector used to construct small insert metagenomic libraries; kan^r^ | Invitrogen |
| pHP45Ω | Vector containing the spectinomycin resistance cassette used for mutagenesis of *E. coli acuI* (*yhdH*) in J557; spec^r^ | Prentki and Krisch (1984) |
| pBIO1729 | *Halomonas* sp. HTNK1 *acuK* and *acuN* genes cloned in pBluescript; amp^r^ | Todd *et al*. (2010) |
| pBIO1879 | Derivative of pK19*mob*, used to mutagenise *S. fredii* NGR234; kan^r^, spec^r^ | Todd *et al.* (2011) |
| pBIO2011 | *E. coli* BW25113 *yhdH* (*acuI*) gene cloned in pET21a | Todd *et al*. (2012) |
| pBIO2079 | Wastewater treatment plant metagenomic library cosmid containing *acuI*-like gene in pLAFR3; tet^r^ | This work |
| pBIO2081 | Wastewater treatment plant metagenomic library cosmid containing *acuI*-like gene in pLAFR3; tet^r^ | This work |
| pBIO2151 | Cast water biofilm metagenomic library plasmid containing *acuI*-like gene in pCR-XL-TOPO; kan^r^ | This work |
| pBIO2152 | Fly compost metagenomic library plasmid containing *acuI*-like gene in pCR-XL-TOPO; kan^r^ | This work |
| pBIO2153 | Cast water biofilm metagenomic library plasmid containing *acuI*-like gene in pCR-XL-TOPO; kan^r^ | This work |
| pBIO2154 | Cast water biofilm metagenomic library plasmid containing *acuI*-like gene in pCR-XL-TOPO; kan^r^ | This work |
| pBIO2155 | Cast water biofilm metagenomic library plasmid containing *acuI*-like gene in pCR-XL-TOPO; kan^r^ | This work |
| pBIO2160 | Fly compost metagenomic library plasmid containing *arkA* gene in pCR-XL-TOPO; kan^r^ | This work |
| pBIO2167 | *arkA* gene from pBIO2160 subcloned in pBluescript | This work |
| pBIO2170 | *Novosphingobium tardaugens* ARI-1 library cosmid containing *bau*/*vut* genes; tet^r^ | This work |
| pBIO2173 | *S. fredii* NGR_c37150 (*vutD*) cloned in pBluescript; amp^r^ | This work |
| pBIO2174 | *S. fredii* NGR_b20860 (*vutE*) cloned in pRK415; amp^r^ | This work |
| pBIO2176 | *S. fredii* NGR_c37150 (*vutD*) internal fragment cloned in pBIO1879; kan^r^, spc^r^ | This work |
| pBIO2177 | *S. fredii* NGR_b20860 (*vutE*) internal fragment cloned in pBIO1879; kan^r^, spc^r^ | This work |
| pBIO2195 | *Bacillus megaterium* DSM 319 gene BMD_3924 (*arkA*) cloned in pET21a; amp^r^ | This work |

References:

Baba T, Ara T, Hasegawa M, Takai Y, Okumura Y, et al. (2006) Construction of *Escherichia coli* K-12 in-frame, single-gene knockout mutants: the Keio collection. Mol Syst Biol 2: 2006.0008.

Figurski DH, Helinski DR (1979) Replication of an origin-containing derivative of plasmid RK2 dependent on a plasmid function provided *in trans*. Proc Natl Acad Sci USA 76: 1648-1652.

Fujii K, Satomi M, Morita N, Motomura T, Tanaka T, et al. (2003) *Novosphingobium tardaugens* sp. nov., an oestradiol-degrading bacterium isolated from activated sludge of a sewage treatment plant in Tokyo. Int J Syst Evol Microbiol 53: 47-52.

Keen NT, Tamaki S, Kobayashi D, Trollinger D (1988) Improved broad-host-range plasmids for DNA cloning in gram-negative bacteria. Gene 70: 191-197.

Prentki P, Krisch HM (1984) *In vitro* insertional mutagenesis with a selectable DNA fragment. Gene 29: 303-313.

Simon R, Quandt J, Klipp W (1989) New derivatives of transposon Tn*5* suitable for mobilization of replicons, generation of operon fusions and induction of genes in gram-negative bacteria. Gene 80: 161-169.

Staskawicz B, Dahlbeck D, Keen N, Napoli C (1987) Molecular characterization of cloned avirulence genes from race 0 and race 1 of *Pseudomonas syringae* pv. glycinea. J Bacteriol 169: 5789-5794.

Todd JD, Curson ARJ, Kirkwood M, Sullivan MJ, Green RT, et al. (2011) DddQ, a novel, cupin-containing, dimethylsulfoniopropionate lyase in marine roseobacters and in uncultured marine bacteria. Env Microbiol 13: 427-438.

Todd JD, Curson ARJ, Nikolaidou-Katsaraidou N, Brearley CA, Watmough NJ, et al. (2010) Molecular dissection of bacterial acrylate catabolism - unexpected links with dimethylsulfoniopropionate catabolism and dimethyl sulfide production. Env Microbiol 12: 327-343.

Todd JD, Curson ARJ, Sullivan MJ, Kirkwood M, Johnston AWB (2012) The *Ruegeria pomeroyi* *acuI* gene has a role in DMSP catabolism and resembles *yhdH* of *E. coli* and other bacteria in conferring resistance to acrylate. PLoS One 7: e35947.

Trinick MJ (1980) Relationships amongst the fast-growing rhizobia of *Lablab purpureus*, *Leucaena leucocephala*, *Mimosa* spp., *Acacia farnesiana* and *Sesbania grandiflora* and their affinities with other rhizobial groups. J Appl Bacteriol 49: 39-53.

Wood WB (1966) Host specificity of DNA produced by *Escherichia coli*: bacterial mutations affecting the restriction and modification of DNA. J Mol Biol 16: 118-133.

Yanisch-Perron C, Vieira J, Messing J (1985) Improved M13 phage cloning vectors and host strains: nucleotide sequences of the M13mp18 and pUC19 vectors. Gene 33: 103-119.

Young JPW, Crossman LC, Johnston AWB, Thomson NR, Ghazoui ZF, et al. (2006) The genome of *Rhizobium leguminosarum* has recognizable core and accessory components. Genome Biol 7: R34.
