# Supplementary material for: Screening of Metagenomic and Genomic Libraries Reveals Three Classes of Bacterial Enzymes That Overcome the Toxicity of Acrylate
Source: PLoS One. 2014 May 21;9(5):e97660. doi: 10.1371/journal.pone.0097660 (PMC4029986; doi:10.1371/journal.pone.0097660)
Supplement: Table S11 — Oligonucleotide primers used in this study. (DOCX) [file pone.0097660.s011.docx]

**Table S11. Oligonucleotide primers used in this study.**

| **Primer** | **Sequence (5’ to 3’)^1^** | **Used for** |
| --- | --- | --- |
| yhdHspecredFOR1 | GAAATTATTTTCCAATCCATCATGCACATGAGGACCACTTATCGTGCCGTGATCGAAATC | Amplifying spectinomycin cassette with *E. coli acuI* flanking sequences |
| yhdHspecredREV1 | TCAAAATGAGTTCCCTCTCTTATTATTCCCTGCTAAATGGTCATCCACCGGATCAGCTTAGTA | Amplifying spectinomycin cassette with *E. coli acuI* flanking sequences |
| yhdHEXTFOR1 | GAAATTATTTTCCAATCCATCATGC | Checking for the disruption of the *acuI* (*yhdH*) gene in *E. coli* strain J557 |
| yhdHEXTREV1 | TCAAAATGAGTTCCCTCTCTTATTATTCC | Checking for the disruption of the *acuI* (*yhdH*) gene in *E. coli* strain J557 |
| specINTFOR1 | GCCCGTTCCATACAGAAGCTGG | Checking for the disruption of the *acuI* (*yhdH*) gene in *E. coli* strain J557 |
| specINTREV1 | CCAATTTGTGTAGGGCTTATTATGC | Checking for the disruption of the *acuI* (*yhdH*) gene in *E. coli* strain J557 |
| 366NPDXbaFOR1 | GGATTACTCTAGATGTACTGTATTAAACG | Cloning pBIO2160 *arkA* in pBIO2167 |
| 366NPDBamREV1 | CGAACGAGTAGTTGACGGATCCAAGC | Cloning pBIO2160 *arkA* in pBIO2167 |
| C37150XbaFOR1 | CGCCCGTCTAGAGCTTGCCGGAATCG | Cloning NGR_c37150 (*vutD*) in pBIO2173 |
| C37150PstREV2 | GCGTTTCAAACTGCAGGCGGCGTATAGC | Cloning NGR_c37150 (*vutD*) in pBIO2173 |
| b20860HindFOR1 | CGGCGTCGAGAAGCTTGTCAGGGACC | Cloning NGR_b20860 (*vutE*) in pBIO2174 |
| b20860BamREV1 | CGATAAAGGGGATCCTTGTCATCTTGC | Cloning NGR_b20860 (*vutE*) in pBIO2174 |
| 37150pK19XbaF2 | GCTGAATTCTAGACTGATGCGTGAGC | Cloning internal fragment of NGR_c37150 (*vutD*) in pBIO2176 |
| 37150pK19PstR1 | CCTTCTGGTCTGCAGTGGCGAACAGCGC | Cloning internal fragment of NGR_c37150 (*vutD*) in pBIO2176 |
| 20860pK19XbaF1 | GGTGCGATCTAGAGGCTGCGGCTCAACC | Cloning internal fragment of NGR_b20860 (*vutE*) in pBIO2177 |
| 20860pK19EcoR1 | CCCTCGTAGAATTCCGGATTGGAGAGC | Cloning internal fragment of NGR_b20860 (*vutE*) in pBIO2177 |
| BacArkANdeFOR1 | GGGGATACACATATGAAAACACGGG | Cloning *Bacillus megaterium* DSM 319 gene BMD_3924 (*arkA*) in pBIO2195 |
| BacArkABamREV1 | GCTCGGATCCTTTTCATTCTCTCACC | Cloning *Bacillus megaterium* DSM 319 gene BMD_3924 (*arkA*) in pBIO2195 |
| M13F | CGCCAGGGTTTTCCCAGTCACGAC | Universal forward primer for end-sequencing of pBluescript-, pRK415- and pBIO1879-based clones, and pLAFR3 and pCR-XL-TOPO library cosmids and plasmids |
| M13R | TCACACAGGAAACAGCTATGAC | Universal reverse primer for end-sequencing of pBluescript-, pRK415-based clones, and pLAFR3 and pCR-XL-TOPO library cosmids and plasmids |
| lacZ | GCCAGCTGGCGAAAGGGGGATGTGC | Used to locate Tn*5lacZ* insertions in mutated derivatives of pBIO2170 |

^1^underlined sequences indicate restriction sites introduced into primers
